# Efficacy Evaluation of a Case-Specific Approach for Surgical Treatment of Inicisional Ventral Hernia

**DOI:** 10.29337/ijsp.147

**Published:** 2021-06-29

**Authors:** Svetlana Sokolova, Andrey Sherbatykh, Konstantin Tolkachev, Vladimir Beloborodov, Vadim Dulskiy, Natalia Kozlova, Vladimir Vorobev

**Affiliations:** 1 Irkutsk State Medical University, Irkutsk, Russian Federation

**Keywords:** Abdominal wall, Endoprosthesis, Quality of life, Surgery, Ventral hernia

## Abstract

**Background:**

The research aims to improve the surgical treatment results of incisional ventral hernia by applying a case-specific approach and a new method of anterior abdominal wall surgery.

**Materials and Methods:**

The paper reports the results of the prospective dynamic cohort study on 219 patients under 60 years of age, with small and medium hernias and up to 10 cm defects in the anterior abdominal wall (W1–W2), who underwent incisional ventral hernia treatment with mesh endoprostheses.

**Results:**

The paper offers a selection algorithm for anterior abdominal wall repair surgery and an original proprietary technique. We have developed and described in detail a new ‘extra-sublay’ technique of surgical intervention. The paper displays the frequency and pattern of complications, as well as the quality of life of patients after different prosthetic surgeries. In the main group, 65.0% of patients showed improvement, 88.4% showed long-term surgical success, 13.6% faced complications, and 4.5% experienced recurrence.

**Conclusion:**

After receiving the “on lay” treatment, 59.4% of patients showed positive results, 74.7% showed long-term surgical success, 40% had complications, and 3.1% experienced recurrence. After the “sub lay” intervention, 40.0% of patients demonstrated excellent results, 81.9% reached long-term success, 12% had complications, and 1.4% encountered recurrence.

**Highlights:**

- The article shows a selection algorithm for anterior abdominal wall plastic repair method.
- One of the factors that cause relapses and ventral hernias themselves is obesity.
- The authors’ method of the VH surgical treatment has shown good results.
- Excellent indicators showed 65.0% of patients of the main group.

## 1. Introduction

The incisional ventral hernias (IVH) treatment currently continues to be quite complex and, unfortunately, surgical procedure is incapable of solving the hernia problem completely. The increasing number of abdominal surgeries leads to an increasing number of IVH. According to different authors, incisional ventral hernias and recurrent abdominal hernias are likely to occur in 2–20% cases after laparotomies and postoperative complications [1,2]. Most often, IVH develops after gynecological surgeries (26–50% incidence) and biliary tract interventions (20–30% incidence) [1,2]. Analysis showed that the first 3 years after laparotomy are the high-risk period for hernia development (92–97% incidence) [3]. The frequency of recurrent hernias after anterior abdominal wall surgery can reach 50% [4,5,6].

Synthetic prostheses in herniology have reduced the frequency of hernia recurrences to 6–10% [7,8,9], but most authors prefer to use combined prosthetic methods, considering them the best option for surgical IVH treatment [7,8,9]. At the same time, the widespread use of prosthetic methods increased the number of wound complications and decreased the quality of life in 30–40% of operated patients [10,11]. In general, the wound complications frequency after anterior abdominal wall surgery for IVH varies from 20.9 to 67% [12,13,14]. The structure of the wound complications mainly indicates: suppuration, the wound edges divergence, seroma, inflammatory infiltrate, long-term lymphorrhea, ligature fistulas, and wound edges necrosis [14,15,16,17]. The “new” complications, those caused exclusively by synthetic mesh prostheses, are the prosthesis migrating into the lumen of the hollow organ; intestinal fistula induced by trauma to the intestinal wall; prosthetic cyst; adhesive intestinal obstruction (seen when the intestine adheres to the prosthesis); and mesh rupture with the recurrent hernia or infringement of the hollow organs [14,18,19]. However, data collected about these complications are contradictory.

The choice of the surgery method for IVH, especially the recurrent ones, is also somewhat difficult. The traditional “sub lay” surgery is a complex, long-term, and traumatic process that is hard to perform amid a pronounced cicatricial adhesion of the anterior abdominal wall tissues. It may cause damage (a.a. epigastric superior et inferior) with the atrophy development and rectus muscles scarring, as well as hematomas and fluid formations, prolonged pain syndrome.

Dissatisfaction with the results of the standard anterior abdominal wall surgery makes surgeons seek ways to improve them not only by developing and implementing new methods but also by a using a differentiated approach to the choice of the surgery method. At the same time, the IVH surgical treatment should be minimally invasive and provide not only reconstruction of the anterior abdominal wall with maximum function recovery, but also reduction of the frequency of wound complications and hernia recurrences, while maintaining a high level of the quality of life of operated patients [20,21,22].

The current research aims to analyze the effectiveness of surgical IVH treatment under a differentiated approach by looking at the immediate and long-term results of various anterior abdominal wall surgery methods.

## 2. Material and Methods

The authors declare that the work is written with due consideration of ethical standards. The Human Experiments Ethics Committee of XXXX University (Protocol XXXX) approved the study based on ethical principles. The research is being reported in line with STROCSS criteria.

To choose the method of anterior abdominal wall surgery, a differentiated approach was used. We examined patient’s age, comorbidity status, hernia size, and hernia duration. The examination was performed with the patient in a supine position with the neck flexed toward the sternum and standing. Attention was paid to the localization of the hernial protrusion, its shape, size. If a hernia is discovered on palpation, the following measures are documented: shape, size, flexibility or incorrigibility, and the size of the hernia gate. Among the instrumental methods used were X-ray examination of the digestive tract, spirography, electrocardiography (ECG), ultrasonography (USG), and computed tomography (CT). The examination aimed to detect concomitant pathologies, adhesions, and additional hernia gates. Ultrasonography and CT examinations revealed 3 types of integrity violations in the aponeurosis:

1. Hernial defect in the midline at the medial edges of the rectus muscles. The muscles were separated due to the formation of a hernia and the destruction of the white line. No damage to the structure of the muscles has been detected.
2. Destruction of the white line and damage to the muscles and aponeurotic sheaths of the lateral muscles of the anterior abdominal wall.
3. Combines the previous symptoms and a defect in the aponeurosis larger than 15 cm.

### 2.1. Patients

The study reports the results of the surgical treatment of 219 patients with small and medium-sized hernias who underwent prosthetic operations with polypropylene mesh (PPM) for incisional median ventral hernias in the XXXX Department of XXXX Hospital from XXXX to XXXX.

There were three groups of patients according to the position of the synthetic prosthesis: the main group and two comparison groups. The main group (MG) included 52 patients (23.7 ± 2.6%) who underwent anterior abdominal wall plastic surgery via the proposed “extra-sublay” method. The first clinical comparison group (CCG I) had 95 patients (43.4 ± 3.8%) operated by the “on lay” method. The second clinical comparison group (CCG II) consisted of 72 patients (32.9 ± 3.5%) who were operated by the “sub lay” method. All surgical interventions were performed using a mesh polypropylene endoprosthesis Lintex-Esfil (Saint Petersburg).

Patients under 60 years of age, with 10-cm defects in an anterior abdominal wall (W1–W2) and smaller, without severe comorbidities were assigned to the “on lay” allopasty.

Patients aged 60 and older, with comorbidities, with an increased risk of developing complications and recurrences, and with 10 to 20-cm defects in an anterior abdominal wall (W3–W4) were exposed either to a “sub lay” method or to an “extra-sublay” surgery. The method was used for aponeurosis defects of types 2 and 3.

Contraindications to recurrent hernia surgery were decompensated cardiovascular or respiratory comorbidities; pregnancy; large hernias in elderly patients with flaccid anterior abdominal wall; poor preoperative preparation.

Preoperative preparation has the following steps:

1. Compensation for concomitant pathology.
2. Preparation of the abdominal cavity for the insertion of fallen organs.
3. Preparation of the digestive tract.

The insertion of organs that have fallen into a reduced abdominal cavity is a crucial step because the respiratory regime, as well as the activity of the cardiovascular system, changes during the postoperative period. Preparation of the digestive tract includes cleansing the intestines of the contents with laxatives and enemas.

The second surgical treatment was performed no earlier than 8–12 months following the first intervention. The postoperative treatment involved the prevention of heart and lung disorders, intestinal paresis and bloating, thromboembolic complications, and wound suppuration.

### 2.2. The treatment technique

In the preoperative period, patients with IVH underwent a comprehensive examination, including clinical, laboratory, and instrumental methods. All patients underwent preoperative antibiotic prophylaxis, thromboembolic complications prevention, which continued after the operation, along with adequate pain relief therapy and concomitant pathology correction. All surgical interventions include intubation (endotracheal) anesthesia.

**Figure 1:**
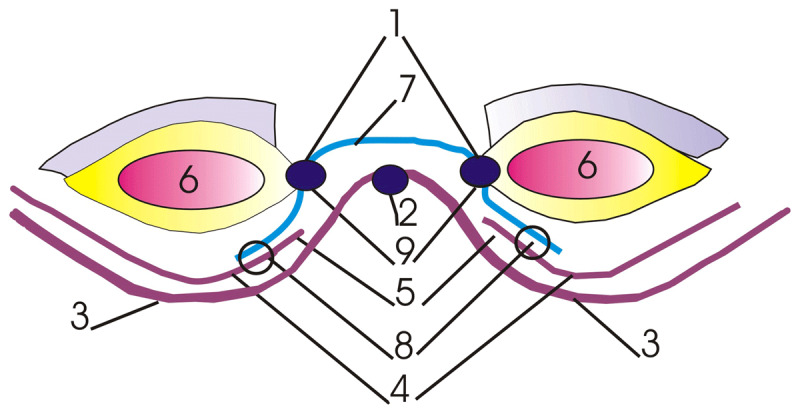
Method “extra-sublay” for incisional ventral hernias surgery, where: 1 – hernia defect edge, 2 – hernial sac, 3 – peritoneum, 4 – transverse fascia, 5 – aponeurosis, 6 – abdominal muscles, 7 – polypropylene mesh, 8 – single knot sutures, 9 – continuous suture.

Since 2010, the surgical department of XXXX Hospital has been using an original method of anterior abdominal wall surgery — the “extra-sublay” technique (***Figure 1***).

The extra-sublay surgical technique involves the following steps:

1. Cutting the anterior abdominal wall.
2. Excising the scar, excess skin, and fat. After excision of the old postoperative scar and mobilization of aponeurosis from the subcutaneous tissue at a distance of 4 cm from the hernial gate edge, selecting the hernial sac to open, trying to preserve its flap as much as possible.
3. Cutting out the hernia sac completely or partially.
4. Separating the transverse fascia by 1.5–2 cm from the edges of the hernia defect edge. Next, sewing the hernia gate edges together with separate nodal seams under the flap of the hernia sac.
5. Placing a polypropylene mesh in the formed pocket between the separated transverse fascia and the aponeurosis to the entire depth of the isolated transverse fascia along the entire perimeter of the hernia defect edge. We covered the seam line with a mesh implant, fixing it along the perimeter to the aponeurosis of the anterior abdominal wall muscles with a continuous seam. The size of the implant should be big enough to keep its outer edges at least 4 cm away from the aponeurosis suture line. From the flap side in the mesh prosthesis at a distance of 2 cm from the aponeurosis suture, we made a longitudinal incision, the so-called “window” to output the hernial sac flap. Along the incision line, we fixed the hernia sac to the mesh with separate U-shaped seams to eliminate the “window”. The peritoneum flap we sew on one side along the seam line of the nearest edge of the mesh with additional nodal seams and the free part of the flap covered the entire mesh implant, fixing it with separate nodal seams along the perimeter and throughout the entire area of the endoprosthesis. Thus, the implant was located between the hernial sac and the aponeurosis.
6. Fixing the grid with single nodal sutures to the transverse fascia. To prevent the peritoneal fluid exudation and its accumulation in the subcutaneous tissue, we performed the peritoneal mesothelium desquamation with a gauze buffer and the hernial sac peritoneodesis. It was proved that the peritoneum, devoid of mesothelial cover, loses the ability to produce fluid. A peritoneodesis of the hernial sac, due to the tight fit of the de-epithelized peritoneum to the implant surface, prevents the “dead spaces” formation and the exudate accumulation in the wound [23,24].
7. Fixing a grid to the hernia edge defect by a continuous seam without tension. The operation ends with layer-by-layer suturing of the wound, with drainage of subcutaneous tissue by Redon.

During the postoperative period, the early mobilisation of patients was encouraged. They were allowed to wear an elastic bandage starting from day 2, which remained even after discharge from the hospital. We removed the Redon drains when the amount of liquid was 10.0 ml/day. To monitor the course of the wound process, we used the ultrasound examination on 5–7 days of the postoperative period. In the absence of active complaints and wound complications, with normal laboratory control parameters, patients came home from the hospital under the supervision of a doctor.

### 2.3. Evaluating methods for immediate and long-term results of anterior abdominal wall prosthetics surgery

The effectiveness of the applied methods was determined based on the immediate and long-term results of the prosthetic anterior abdominal wall surgery. At the early follow-up, attention was paid to the postoperative wound drainage timing, as well as the number and nature of wound complications. At the long-term follow-up, we assessed the quality of life and the number of recurrences 6 months to 3 years after surgery. The quality of life assessment was performed via the MOS SF-36 survey. The MOS SF-36 assesses the patient’s physical abilities on a 5-point scale. In our study, physical well-being was the main indicator of the quality of life. The following criteria were used to evaluate it: health status before and after surgery; limited or complete exercise at home and recovery of professional activity; discomfort and pain after surgery; violation of bowel functions (constipation) and breathing (shortness of breath) associated with the operation; wearing a bandage; recurrent hernia. The higher the score, the higher the patient’s quality of life. A score of 43 is rated as excellent, 34–43 being good, 24–33 fair, and 13–23 unsatisfactory (this score also indicates the recurrence of hernia).

### 2.4. Statistics

The Student’s t-test and the Mann-Whitney (U) test were used to evaluate the difference in quantitative characteristics. For qualitative indicators, we used the Chi-square criterion (χ^2^). The two-sided Fischer’s method (φ) was used in four-field tables. The differences were considered significant at p < 0.05. Statistical data were processed using the Statistica 6.0 software (StatSoft, USA) and the MS Excel 2010 software.

## 3. Results

For each group, there were no statistically significant differences regarding gender, age, concomitant pathology nature, size, the hernia location, and disease duration (p > 0.05). The distribution of patients by gender revealed the predominance of female subjects in all groups. The average ages of patients in MG, CCG I, and II were 54.0 ± 6.1, 59.0 ± 9.3, and 56.0 ± 10.2 years, respectively. According to the Chevrel and Rath classification (SWR-classification) (23), patients with hernia gate sizes of 5–10 cm (W2, 155: 70.7 ± 3.7% of all cases) and 10–15 cm (57: 26.0 ± 3.0% of all cases) predominated.

In the CCG I group, the hernial defect was more often localized in the suprapubic (M1; n = 25, or 26.3 ± 4.5%) and peripheral (M2; n = 42, or 44.2 ± 5.1%) observation areas. In the MG and CCG II groups, it was more frequency detected in the peripheral (M2; n = 21, or 40.4 ± 10.5% vs n = 27, or 37.7 ± 6.8%) and sub-umbilical (M3; n = 16, 30.8 ± 9.9% and n = 25, 34.7 ± 6.7%) observation areas. Recurrent hernias (R 1–2) were observed in 41 (18.7 ± 3.0%) of all operated patients. One hundred and six (48.4 ± 3.8%) patients had a duration of this disease of 1 to 3 years. The most common concomitant diseases were those of the cardiovascular system, such as hypertension (n =161, or 73.5 ± 3.4%) and coronary heart disease (n = 67, or 30.6 ± 3.5%). The II–III degree obesity were found in 96 patients (43.8 ± 3.8%). Diabetes was found in 25 patients (11.4 ± 2.4%). ***Table 1*** presents the characteristics of the IVH patients.

**Table 1:** Characteristics of patients with IVH.


PARAMETER	MAIN GROUP (MG) n = 52	p-VALUE	ONLAY(CCG I) n = 95	p-VALUE	SUBLAY (CCG II) n = 72	p-VALUE	t-TEST	χ2

Average age, years	54.00	1.5708E-18	59.00	0.0087	56.00	4.201E-12	0.1278042	0.9186116

Hernia size of 5–10 cm (W1–W2), %	50.00	0.01579077	90.00	0.015	70.00	4.268E-15	0.2210655	0.31140322

Hernia size of 10 to 20 cm (W3–W4), %	50.00	0.01579077	10.00	0.0157	30.00	1.38E-06	0.2851972	0.60653066


When the size of the hernial gate was more than 20 cm (W4) and the anterior abdominal wall tissues were under tension, it was necessary to use a non-tensioned “inlay” alloplasty to prevent the development of intra-abdominal hypertension syndrome.

In case of inability to differentiate anatomically scar-modified tissues of the anterior abdominal wall, a modified alloplasty method was used. It was also the case of patients with the most favorable conditions for the use of a hernia sac: patients under 60 years old with an average hernia size (W2) and a duration of the disease of up to 3 years [25].

***Table 2*** presents the comparison of the proposed technique for ventral hernia surgery with the two most common methods.

**Table 2:** Comparative characteristics of ventral hernia surgery methods.


PARAMETER	MAIN GROUP (MG) n = 52	p-VALUE	ONLAY(CCG I) n = 95	p-VALUE	SUBLAY (CCG II) n = 72	p-VALUE	t-TEST	χ2

Positive outcomes, %	65.00	0.00036065	59.00	0.002	40.00	1.066E-08	0.1320869	0.54378056

Postoperative superficial infection	1	1.5708E-18	3	0.005	4	0.2614641	0.065972	0.24836468

Hematoma formation	3	1.5708E-18	3	0.00016	3	0.3916251	0.089086	0.8824969

Postoperative seroma formation	1	1.5708E-18	2	0.0026	2	0.5724067	0.0537613	0.51341712

Bowel obstruction	0	1.5708E-18	1	0.0001	1	0.801252	1.5708E-18	0.60653066

Wound dehiscence	1	0.45493643	2	0.00015	2	0.5724067	0.0106241	0.2865048

Deep incisional infections	3	1.5708E-18	2	0.00015	5	0.1717971	0.1216834	0.53526143

Organ space infections	0	0.01579077	1	0.00015	1	0.801252	0.0103582	0.36787944

Postoperative hospital stay	10	0.01579077	8	0.000590	10	0.0185661	0.2080663	0.4704958

Long-term outcomes, %	88.00	0.01579077	74.70	0.00015	82.00	1.143E-17	0.00015	0.3211

Complications, %	13.6	0.01579077	40	0.001216	0.12	0.9893338	0.1032602	0.4009

Recurrence	4.5	0.04428011	3.1	0.00015	1.4	0.00185	0.1183936	0.0328


Clinical studies have shown that the timing of postoperative wound drainage, the nature and frequency of wound complications, and the quality of life after surgery depend on the method of anterior abdominal wall surgery, namely, the location of the implant. Thus, the period of subcutaneous tissue drainage in the MG (4.09 ± 0.79 days) and CCG II (3.72 ± 1.26 days) groups was significantly shorter compared to the CCG I (4.8 ± 1.64 days) group (p 0.05). Starting from day 3, MG patients showed a significant decrease in the discharge liquid rate to 20 (16.5–30) ml/day. By the 5th day of the follow-up, it dropped to 10 (6.25–13.75) ml/day. In the “on lay” group (CCG I), the discharge liquid rate fell to 30 (20–40) ml/day (p < 0.05) and 20 (15–30) ml/day (p < 0.01), respectively. After 3 days, the MG and CCG II groups became comparable in terms of the discharge liquid rate (p > 0.05).

The use of anterior abdominal wall surgery methods with the isolation of endoprosthesis from the subcutaneous tissue allowed reducing the number of wound complications in the early postoperative period. Thus, there were just 3 (13.6%) patients in the MG group suffering from wound complications, 6 (12%) in the CCG II group, and a total of 38 (40.0%) patients in the CCG I group (χ2 = 4.6583, p < 0.05; χ2 = 10.1484, p < 0.01). The most common complication was fluid accumulation in the subcutaneous tissue, caused by the contact of subcutaneous tissue with the endoprosthesis, and the development of ischemia of the skin and deep layers of subcutaneous tissue after their wide mobilization and in the absence of dense contact with aponeurosis. Therefore, postoperative wound seromas were more common after “on lay” plastic surgery in the CCG I group (32 cases, or 33.7%) than in the MG (2 cases, or 9.1%) (χ2 = 4.1163, p = 0.04) and CCG II (4 cases, or 8%) groups (χ2 = 10.1484, p < 0.01). We considered all liquid formations detected via ultrasound. There were no significant differences in the frequency of other complications (p > 0.05).

To monitor the course of the wound process, all patients underwent an ultrasound examination of the postoperative wound between days 5 and 7 after surgery. Patients with a large volume of liquid accumulations and their clinical manifestation underwent dotted exudate under ultrasound control. The number of punctures depended on the dynamics of the process and was averaged 1–3 punctures. The amount of extravasate less than 10.0 ml, the disappearance or reduction of the size of the liquid formation were the criteria for completion of punctures. The number of patients in the CCG I group who required a puncture reached 4 (4.2%), whereas in the CCG II group, there were just 2 (4.0%) such patients. In the MG group, just 1 (4.5%) patient needed punctual management of fluid formation. In all cases, there was a positive result. In the CCG I group, one patient (1.05%) had the spontaneous opening of seroma dehiscence, so had one patient (2%) in the CCG II group. The CCG I group had two (2.1%) cases of subcutaneous tissue hematoma, while the CCG II group and the MG group both had one such case recorded (2% and 4.5%, respectively). The cause of hematomas was inadequate hemostasis during surgery.

After “on lay” surgery, postoperative wound suppuration occurred in one patient (1.05%) in the CCG I group. There were also one case of wound infiltration (1.05%) and one case of ligature fistula of the anterior abdominal wall (1.05%) in this group. The fistula needed excision with the removal of the anterior abdominal wall section.

We studied the long-term outcomes of the operation, including the quality of life and hernia recurrence rates. There were 176 patients at the long-term follow-up: 71 (74.7%) in the CCG I group, 59 (81.9%) in the CCG II group, and 46 (88.4%) in the MG group.

After “on lay” plastic surgery, recurrences occurred in 3 patients (3.1%) in the CCG I group, 2 of whom refused to re-operate. The third patient underwent surgery in another medical facility a few months after the primary intervention. The reason for the recurrence was the inadequate size of the mesh implant and insufficient fixation of the upper edge of the mesh. In the CCG II and MG group, recurrence occurred in one case (1.4% and 4.5%, respectively). There were no significant differences in the recurrence frequency between the groups (p > 0.05).

According to the results of the Quality of Life survey, the percentage of patients with excellent quality of life in the MG (65.0%) and CCG II (59.4%) groups was higher than in the CCG I group (40.0%, RF < 0.01). The average quality of life scores of the MG and CCG II groups were 44.7 ± 7.6 (79.8%) and 45.4 ± 6.8 (80.9%), respectively, whereas the CCG I group scored significantly lower (p < 0.01); the average quality of life score of the CCG I group was 40.6 ± 6.3 (72.4%) points, indicating good quality of life. The main factors that harmed the quality of life were the restriction and reduction of the daily and professional activities (38 cases, or 40%). The frequency of this factor in the CCG I group was significantly higher compared other groups (RF < 0.05).

## 4. Discussion

The proposed IVH treatment methodology consists of fixing the polypropylene mesh & hernia defect edge with additional sutures; this particular technique yielded better results compared to other two widely used approaches of plastic surgery, “onlay” and “sublay”, and led to fewer recurrences. However, the frequency of recurrence was heavily dependent on the hernia size as well as the patient’s ability to be cautious in the postoperative regimen. In [1,5], the authors point out that the surgical intervention is performed by separating the components by posterior opening or using an endoscopic approach, which shows good results and eliminates the risk of recurrences and blood loss, but this tactic involves massive surgical intervention and has contraindications for certain categories of patients, for example, the geriatrics. The retro rectus method with the polypropylene mesh, proposed in [5,18,19] for restoring the midline of the anterior abdominal wall with IVH, according to the results presented by the authors, is safe and characterized by minimal complications and recurrences for medium-sized hernias (6–13%). For the recovery of large sizes hernias, the method is recognized as the procedure of choice. In large hernias, there is a possibility of using the patient’s tissues with tensor fascia lata and vastus lateralis to close the abdominal wall [17]. The quality of the patient’s life after surgery and the risk of complications and recurrences are also affected by the quality of the mesh material, but the presented reviews do not indicate the brands of materials and their impact on the result [17]. The quality of the mesh and its composition directly affect the interaction of the mesh with the tissue, the encapsulation of the mesh, as well as the risk of infection, which was 1% in our studies. We used the sutures method for fixing tissues with mesh, but the results of using special fixing devices during the operation were presented also. For example, [2] indicates postoperative pain syndrome and the need to use painkillers up to 12 weeks after surgery, since the fixing nails are made of polydioxanone, hydrolysis of which is completed within 12–18 months after implantation. Special materials used for fixing meshes, for example, ProGrip^TM^ according to the authors [11] showed a satisfactory (average) fixing strength of 1.3 N/cm (±STE 0.2). The strength of the mesh fixation with the Tisseel^TM^ solution was 2.6 N/cm (±STE 0.5). According to the authors [7,11], LifeMesh^TM^ adhesive had an average fixing strength of 8.0 N/cm (±STE 2.1). Undoubtedly, the immediate strength of fixing the mesh with adhesive solutions significantly increases the effectiveness of the surgical intervention, but it has remote negative consequences and can cause early recurrence and mesh reduction. However, this fixing method is one of the most non-traumatic methods and demonstrates clear advantages of LifeMesh^TM^ technology [6,7,11]. The authors’ method tested on the main group had clear advantages over the method presented in [11], since it excluded mesh reduction and subsequent complications. The research results also showed the possibility of preventing exudate accumulation in the subcutaneous tissue. Desquamation of mesothelium and peritonitis hernia sac also prevents fluid formation and accumulation in the wound. Our research correlates with the results presented in [24].

In the study [3], which included 64 patients from 25 to 98 years who underwent abdominal surgery, the risk of the complications ranged from 31.2% to 40.6% (26/64), hernia recurrence and mortality were at 7.8%. In our research, there were no patient deaths in the three groups, and the complication rate did not exceed 40%. The developed method of the IVH surgical treatment caused complications in 13.6% of cases, the “sub lay” method applied in the second clinical comparison group caused complications in 12% of cases. Intra-operative fluorescent angiography with indocyanine green can help to prevent recurrences and complications during ventral hernia surgery [15]. Obesity is the single most important reason, which is responsible for causing recurrences, complications, and ventral hernias in its entirety. The authors of the studies [1,6,7,8] presented the treatment results of patients with obesity, which is not only a risk factor for complications during abdominal surgery. The research focused in particular on the complications in the ventral abdominal wall hernias treatment, postoperative hernias, and complications after ventral hernia repair. Obesity also increases the risk of hernia injury and recurrence after treatment. The authors believe that the laparoscopic approach to the IVH treatment minimizes the risks of the complications caused by obesity during abdominal wall surgery. The study [9] demonstrates analogous results in patients with grade III obesity compared to patients with a BMI of ≤39.9 kg/m^2^. It should be noted that the bulk of patients with complications and recurrences (74.5%) who took part in our research were patients with some degree of obesity.

From predicting the clinical and economic results of the IVH treatment throughout life, the developed method is not only comparative in terms of quality and reproducibility of results with other studies but also has some advantages. In a typical group of patients with a ventral hernia, laparoscopic recovery [9] at the time of diagnosis was very cost-effective compared to other interventions commonly used in healthcare in other countries. The average cost of the operation in the US was **$**27,700 … **$**50,000. Patients with a high risk of perioperative mortality, complications, and recurrences need conservative monitoring [9,12]. Due to economic issues, patients who experience no reduction in quality of life caused by ventral hernia also need monitoring [12,15]. Besides, the issue of performing open or laparoscopic operations to eliminate ventral hernias is based on the optimal ratio of the cost of surgery and the quality of life [12,23,24].

## 5. Conclusion

The ventral hernias were treated postoperatively under three approaches, namely, onlay, sublay and extra-sublay. Using a differentiated approach to selecting a plastic surgery method for the treatment of the anterior abdominal wall, as well as deploying the “extra-sublay” plastic surgical method, allowed us to improve the treatment results. The wound complications frequency decreased to 13.6%, the frequency of the liquid form in the subcutaneous tissue fell to 9.1%, and the quality of life after surgery increased to 7.4%. Of all the patients operated, excellent quality of life was reported by 65.0% of patients in the MG group, 59.4% in the CCG I group, and 40.0% in the CCG II group. The long-term success was observed in 88.4% of patients in the MG group, 74.7% of patients in the CCG I group, and 81.9% of patients in the CCG II group. The recurrence rate was 4.5% in the main group, 3.1% in the first clinical comparison group, and 1.4% in the second group. The proposed EXTRA-SUBLAY method allows strengthening the anterior abdominal wall and preventing the formation of accumulations of serous fluid. This technique is recommended for the treatment of complex cases and hernia defect types W3 and W4.

## Data Accessibility Statement

All data will be available on request.
